# Photosynthetic Response of Soybean to Microclimate in 26-Year-Old Tree-Based Intercropping Systems in Southern Ontario, Canada

**DOI:** 10.1371/journal.pone.0129467

**Published:** 2015-06-08

**Authors:** Xiaobang Peng, Naresh V. Thevathasan, Andrew M. Gordon, Idris Mohammed, Pengxiang Gao

**Affiliations:** 1 Department of Biological and Medical Engineering, Shangluo University, Shangluo, Shaanxi, China; 2 School of Environmental Sciences, University of Guelph, Guelph, Ontario, Canada; 3 College of Forestry, Northwest A&F University, Yangling, Shaanxi, China; Glasgow University, UNITED KINGDOM

## Abstract

In order to study the effect of light competition and microclimatic modifications on the net assimilation (NA), growth and yield of soybean (*Glycine max* L.) as an understory crop, three 26-year-old soybean-tree (*Acer saccharinum* Marsh., *Populus deltoides X nigra*, *Juglans nigra* L.) intercropping systems were examined. Tree competition reduced photosynthetically active radiation (PAR) incident on soybeans and reduced net assimilation, growth and yield of soybean. Soil moisture of 20 cm depth close (< 3 m) to the tree rows was also reduced. Correlation analysis showed that NA and soil water content were highly correlated with growth and yield of soybean. When compared with the monoculture soybean system, the relative humidity (RH) of the poplar-soybean, silver maple-soybean, and black walnut-soybean intercropped systems was increased by 7.1%, 8.0% and 5.9%, soil water content was reduced by 37.8%, 26.3% and 30.9%, ambient temperature was reduced by 1.3°C, 1.4°C and 1.0°C, PAR was reduced by 53.6%, 57.9% and 39.9%, and air CO_2_ concentration was reduced by 3.7μmol·mol^-1^, 4.2μmol·mol^-1^ and 2.8μmol·mol^-1^, respectively. Compared to the monoculture, the average NA of soybean in poplar, maple and walnut treatments was also reduced by 53.1%, 67.5% and 46.5%, respectively. Multivariate stepwise regression analysis showed that PAR, ambient temperature and CO_2_ concentration were the dominant factors influencing net photosynthetic rate.

## Introduction

Agroforestry, a conservation land management practice where trees, agricultural crops, grasses and/or animals are grown simultaneously on the same landscape, is being promoted as an alternative management system that can diversify income and improve environmental quality and environmental benefits on the farm landscape [1, 2]. These land-use systems provide various products and benefits for household and national economies including food and medicinal products for humans and animals, timber for construction and fuel, and cash income.

Agroforestry originated in developing countries as a result of high population densities coupled with scarce land resources. Because of the ecological, social and economic benefits associated with agroforestry compared with traditional forestry and agricultural practices, many developing countries and regions have adopted this land-use system [3, 4]. More recently, developed countries in temperate regions (e.g. U.S.A., Canada, Great Britain and Australia) have begun to develop and promote agroforestry systems [5–7]. In southern Ontario, over the past 25 years, investigations on tree-based intercropping systems have revealed the presence of several complementary biophysical interactions associated with this land-use system [8, 9].

Although “tree-influenced microclimatic modifications may act in such a way as to increase the overall productivity of the associated agricultural crop” [9], biophysical interactions are likely to change and become modified as the perennial tree component of the system ages. This study was designed to investigate net assimilation (NA) and photosynthetically active radiation (PAR) associated with an understory soybean crop and microclimatic parameters in three 26-year-old hardwood-based intercropping systems. Our results are compared to those of Reynolds et al. [10] when the same systems were only 12-year-old.

## Materials and Methods

### Site description and plant materials

The 30-ha study site is located at the Guelph Agroforestry Research Station in Wellington County (43°32′28″N Lat., 80°12′32″W Long., elevation 334 m a.s.l.) near Guelph, southern Ontario, Canada. No specific permissions were required for this location and the field studies did not involve endangered or protected species. Eight hardwood and two coniferous species have been annually intercropped with several annual crops at the research site. For this study, soybeans (*Glycine max* L.) were intercropped under 26-year-old hybrid poplar (*Populus deltoides x nigra var* 'DN-177'), silver maple (*Acer saccharinum* Marsh.), and black walnut (*Juglans nigra* L.) and compared to the same systems described by Reynolds et al. [10] when the trees were 12 years old. The tree rows are oriented approximately north and south, with each species planted in groups of eight trees. Tree rows were initially approximately 1 m in width (i.e., 6.7% of the available land area). Soils of the study area are from the Guelph Loam series and the texture ranges from silt loam to loam (Order: Alfisols, group: Typic Hapludalf) [9].

In this study, soybean, a C_3_ species, was intercropped with hybrid poplar, silver maple and black walnut, or grown by themselves in the absence of trees, for a total of four treatments. In the experiment, all tree species chosen were planted with a within-row spacing of 6 m and a between-row spacing of 15 m. The characteristics of selected poplar, maple and walnut trees intercropped with soybeans in 1997 and 2012 (when the trees were 12- and 26-year-old) are given in Table 1.

**Table 1:** 12 and 26-year-old trees (1997 and 2012, respectively) intercropped with soybean at the same study site.

	Poplar		Maple		Walnut^b^
Measurement	1997^a^	2012	1997^a^	2012	2012
Tree height (m)	12.1	19.8	7.6	15.3	14.6
DBH (cm)	22.3	45.2	15.6	31.7	26.0
Depth of live crown (m)	4.9	5.6	2.5	3.0	3.4
Percent canopy closure	43	55	11	16	20

Note: DBH is the diameter at breast height of tree.

^a^ Data of 1997 adapted from Simpson [22].

^b^1997 data not measured.

**Fig 1:**
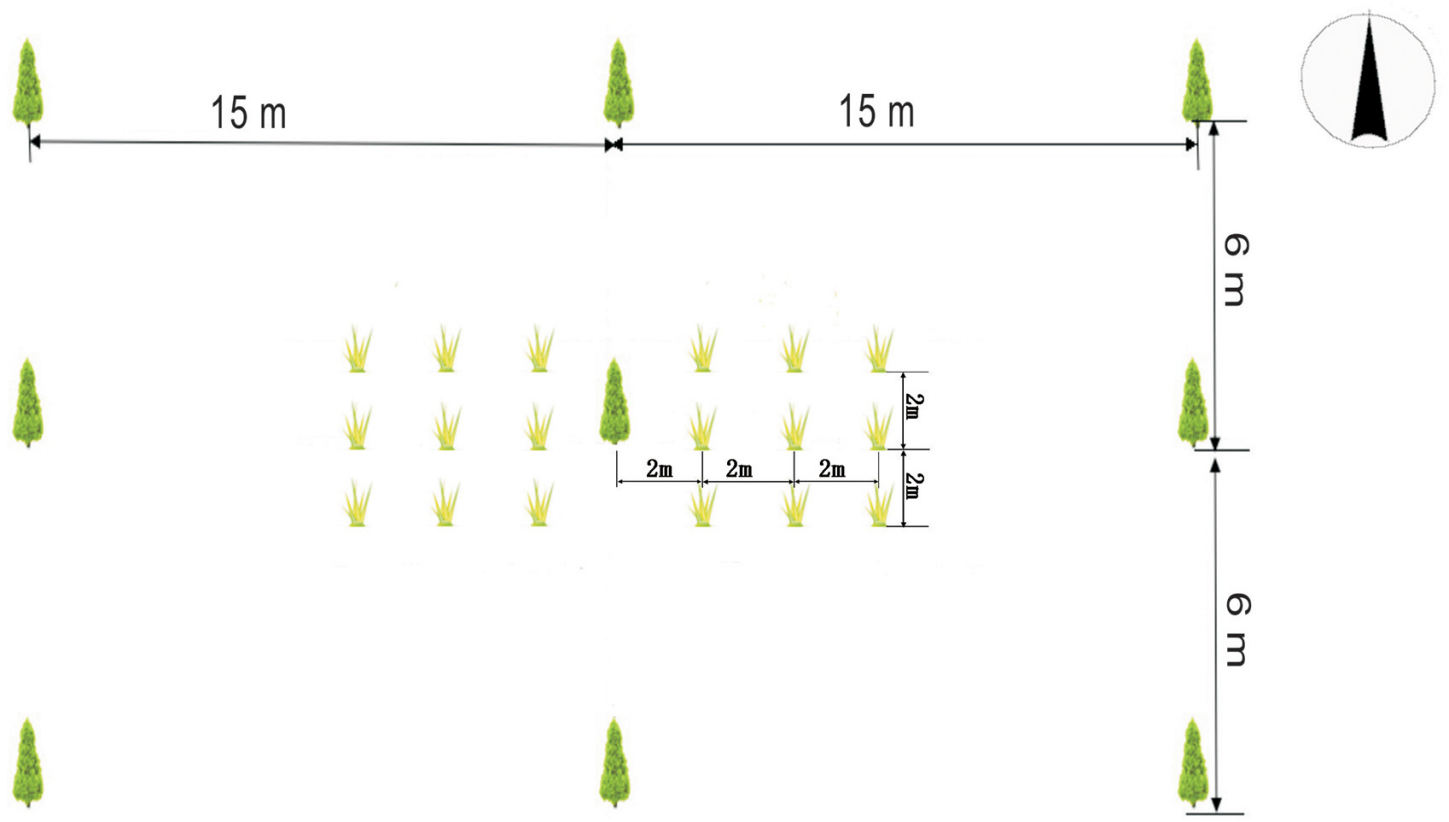
Sampling locations from the tree row into the cropping alley. The crop like symbol refer to sample plot.

Soybeans were grown at a spacing of approximately 0.2 m within rows and 0.3 m between rows for each treatment. For each treatment with trees, three trees were sampled, with 18 sample locations chosen around each tree, at 2 m, 4 m and 6 m east and west of the tree (primary axis perpendicular to the tree row) and at 2 m north and south of each location of the primary axis (Fig 1). For the control treatment, three sample locations were randomly taken.

### Collection and measurement of photosynthetic data

A portable photosynthesis system (LI-6400; LI-COR, Inc., Lincoln, NE, USA) with a red/blue LED light source (LI6400-02B) mounted onto a 6 cm^2^ clamp-on leaf chamber was used to determine NA in sunny and windless weather. A single leaf from the upper canopy of the each soybean was measured at every location (18 in total per tree species) during five clear sunny days between 09:00 and 17:00 at 2 hour intervals in July 2012 (July 4, 5, 6, 8, 10). PAR, ambient temperature, humidity and CO_2_ concentration were also measured concurrently with the LI-6400 Photosynthesis System.

### Soil moisture

Soil was sampled in July, 2012, using a soil auger; soil gravimetric moisture content was measured at a depth of 0 to 20 cm, with soil samples taken from all locations described in Fig 1.

### Measurements of soybean growth, biomass, and yield

A single soybean plant was sampled at each of the 18 locations (i.e. poplar, maple, walnut) and three plants in the control treatment on July 28 and 29 of 2012. A total of 57 soybean plants were harvested. Plants were taken to the lab, where above-ground dry weights, leaf number, plant height, whole plant leaf area and whole plant leaf oven-dried (70°C) weights were determined. Leaf areas were determined using a LI-COR 3100 Leaf Area Meter (LI-COR, Inc., Lincoln, NE, USA).

106 days after planting (15 September 2012), a 1 m^2^ area of soybeans on both sides of the tree rows and in the control treatment were harvested for the determination of aboveground biomass (dry weight basis, 70°C drying temperature and dried for 1 week). Yield was reported on a per hectare basis and does not represent land lost due to tree production.

### Data visualization and analysis

An analysis of variance (ANOVA) was performed as needed, using the statistical software SPSS 12.0.1 for Windows XP. One-way ANOVA’S were performed on soybean data to determine treatment differences for environmental, physiological, and various crop productivity parameters. Within each treatment, differences for crop productivity parameters, daily environmental parameters, and daily physiological parameters for the three major locations (2, 4 and 6 m) were also analyzed using ANOVA. Correlation analysis was performed on mean daily net assimilation, crop growth, biomass, and yield of soybean with environmental and physiological parameters. Multivariate stepwise regression was utilized to investigate the relationship between microenvironmental factors and net assimilation of soybean.

## Results

**Fig 2:**
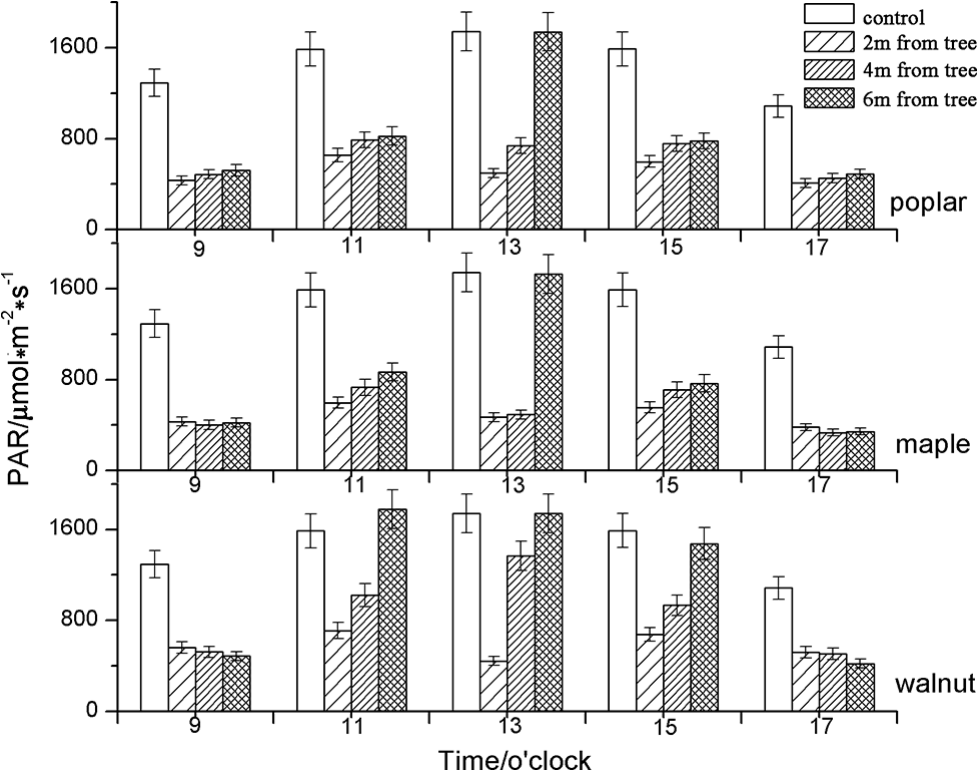
Diurnal PAR for soybean in control plots and within 2 m, 4 m and 6 m of poplar, maple and walnut. Values presented are means (N = 4), error bars indicate the standard error of the mean.

PAR impacting soybean in the different intercropping systems (poplar, maple and walnut) was all lower than that of the control treatment at two hour intervals from 9:00 to 17:00 hours (as shown in Fig 2).

According to Table 2, the total solar radiation reaching the upper parts of soybean canopies in the different intercropping systems (677.3, 614.7, 877.3μmols^-1^ m^-2^) was lower than that in control treatment (1460μmols^-1^ m^-2^). The PAR of all treatments increased with the distance (2, 4 and 6 m) from the tree row (Table 3).

**Table 2:** Differences in measured parameters associated with soybean plants in three intercropping treatments and monoculture control 12 (1997) and 26 (2012) years after establishing poplar, maple and walnut intercropping systems.

Measurement	1997			2012			
	Control	Poplar	Maple	Control	Poplar	Maple	Walnut
PAR (μmol s^-1^ m^-2^)	1525.0a	1251.8a	1301.8a	1460a	677.3b	614.7b	877.3b
Daily net assimilation(μmol m^-2^ s^-1^)	18.6a	14.8a	14.7a	17.73a*	8.31b*	5.76b	9.48b*
Height (cm)	79.1a*	56.5b*	56.9b*	77.7a	28.8b	31.4b	33.4b
Whole plant leaf area (cm^2^)	933.2a*	474.1b*	506.7b*	858.6a	307.2b	317.6b	479.7b
Whole plant leaf weight (gm)	3.6a	1.8b	1.9b	3.56a	1.83b	1.76b	2.00b
Total above-ground biomass (gm)	9.2a	4.6b	5.0b	8.97a	3.06b	3.92b*	4.58b*
Seed yield (tha^-1^)	2.59a	1.50b	1.67b	2.16a	0.61b	0.77b	0.89b

Values are means of (N = 4) 2W, 4W, 6W, 2E, 4E and 6E locations. Within each year, values in each row followed by the same letter are not significantly different (Tukey’s HSD, P<0.05).

* Significant at 10% level.

1997 data adapted from Reynolds [10].

**Table 3:** Within plot microclimate and soybean responses 2, 4, and 6 m from tree row to three 26-year-old hardwood intercropping systems and monocropped soybeans.

Measurement	Control	Poplar			Maple			Walnut		
		2m	4m	6m	2m	4m	6m	2m	4m	6m
PAR (μmol s^-1^m^-2^)	1460a	518b	644a	870b*	486b	533b	825b	582b*	870b	1180a
Daily net assimilation (μmol m^-2^s^-1^)	17.73a	4.14b	7.21b*	13.58a	2.82b*	5.17b	9.29a	3.66b	8.06b	16.71a*
Soil moisture at 20 cm depth (%)	8.458a*	5.026b	5.743b	5.016b	5.698b*	6.478a	6.537b*	5.183b	6.075b	6.265b*
Height (cm)	77.7b	15.5b	33.2b	37.6a	16.7b	35.5b	42.1a	18.6b	37.4b	44.3a
Leaf area (cm^2^) per plant	858.6b	182.3b	331.7b	407.5a	171.1b	255.2b	526.6b	354.8b	386.5b	697.7a
Leaf dry weight (g)/plant	3.56b	0.71b	1.77b	3.01a	0.84b	1.68b	2.77a	0.97b	1.82a	3.22a
Above-ground biomass (g) per plant	8.97a	1.15b	2.29b	5.74b	1.32b	3.5b	6.94a	1.44b	5.12b	7.18a
Seed yield (t/ha)	2.16a	0.39b	0.62b	0.83a	0.26b	0.80b	1.25a	0.48b	0.81b	1.37a

Across all treatments (control, poplar, maple, walnut), values in each row followed by the same letter are not significantly different (Tukey’s HSD, P<0.05).

* Significant at P<0.10 (10%) level.

Compared to the control treatment, the average PAR (2 m, 4 m and 6 m) on soybean under poplar, maple and walnut agroforestry systems was reduced by 53.6%, 57.9% and 39.9%, respectively. The results showed that the reduction of PAR on the understory soybean, as influenced by the different tree species, varied and that the lowest incidence of PAR was found closer to the tree rows. The greatest shading was observed under poplar and maple intercropping systems compared to the walnut system.

**Fig 3:**
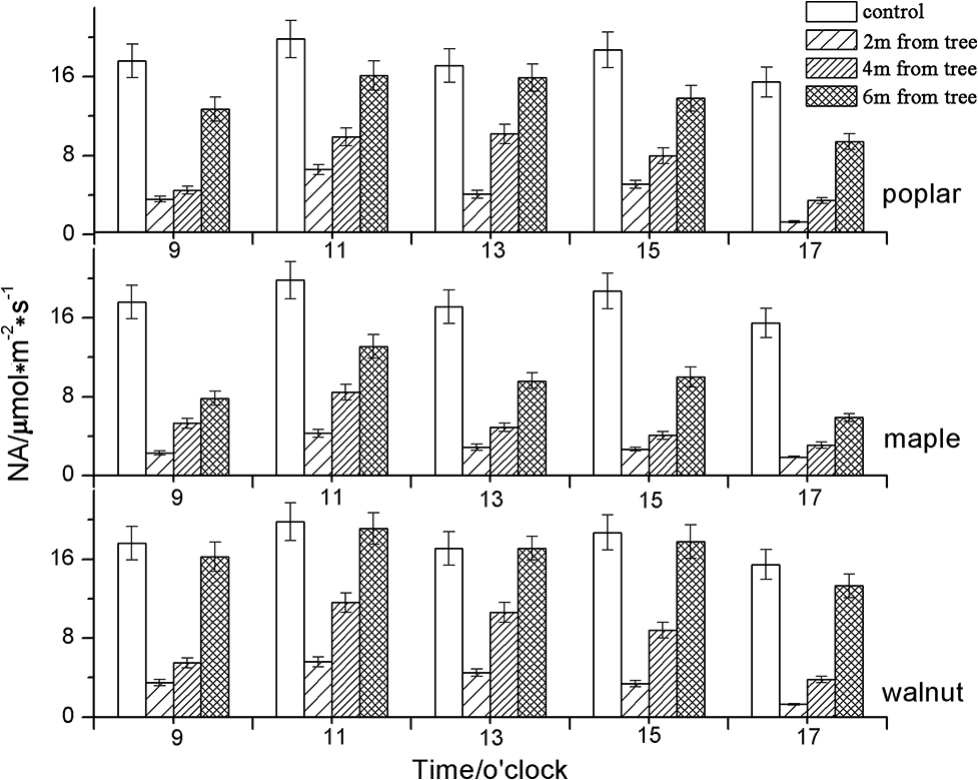
Diurnal NA for soybean in control plots and within 2 m, 4 m and 6 m of poplar, maple and walnut. Values presented are means (N = 4), error bars indicate the standard error of the mean.

The net assimilation of soybean under different treatments (poplar, maple and walnut) was all lower than that in the control treatment at different time periods in the day (Fig 3, Table 2).

The general trend was that the highest net assimilation was at 6 m from the tree row, followed by 4 m and 2 m from the tree row (Table 3). In addition, NA of soybean within different intercropping systems did not show any “midday depression of photosynthesis”, a phenomenon which was observed in the monocropping system between 12 noon and 2 pm. Compared to the control treatment, the average NA of soybean in poplar, maple and walnut treatments was reduced by 53.1%, 67.5% and 46.5%, respectively (Table 3).

Plant height, whole plant leaf area and whole plant leaf weight of soybean in the poplar, maple and walnut treatments were lower than the same parameters measured in the control treatment (Table 2). Similarly, the study found that the total above-ground biomass and seed yield of soybean in different tree-based treatments were lower when compared with the seed yields derived in the monoculture treatment. From 6 m to 2 m from the tree row, the above five parameters decreased when compared to the values derived from the control treatment (Table 3). Compared with the control treatment, the yield of soybean in poplar, maple and walnut treatments was reduced to 71.8%, 64.4% and 58.8%, respectively. The correlation analysis results showed that there was a significant positive correlation between NA and yield (P<0.05).

As shown in Table 3, the soil water content in all three intercropped treatments at 0–20 cm depth was lower than that in the monoculture treatment. There was also a positive correlation between soil water content and soybean aboveground biomass (p<0.05).

When the microclimates of the poplar, maple and walnut intercropping systems were compared to that of the monoculture system (Table 4), the relative humidity was increased by 7.1%, 8.0% and 5.9%, soil water content was decreased by 37.8%, 26.3% and 30.9%, atmospheric temperature was reduced by 1.3°C, 1.4°C and 1.0°C, PAR was reduced by 53.6%, 57.9% and 39.9% and air CO_2_ concentration was also reduced by 3.7μy 3 mol^-1^, 4.27tra mol^-1^ and 2.8amol mol^-1^, respectively.

**Table 4:** Differences in a variety of environmental parameters measured (2012) in three intercropping treatments and monoculture control 26 (2012) years after establishing poplar, maple and walnut intercropping systems.

Treatments	Environmental Parameters				
	PAR μmols^-1^m^-2^	Temperature°C	Relative humidity %	CO_2_ concentration μmol mol^-1^	Soil moisture %
Control	1460.0a	36.57a	48.03b	361.022a	8.458a
Poplar	677.3b	35.32b	51.44a	357.308b	5.262b
Maple	614.7b	35.13b	51.89a	356.837b	6.238b
Walnut	877.3b	35.56b	50.87a	358.236b	5.841b

Values are means of (N = 6) 2W, 4W, 6W, 2E, 4E and 6E locations. Within each measured environmental parameter, values in each column followed by the same letter are not significantly different (Tukey’s HSD, P<0.05).

We used multivariate stepwise regression analysis to determine the relationship between soybean NA (Y variable) and microclimatic factors, where X_1_ is PAR, X_2_ is air temperature, X_3_ is RH, X_4_ is CO_2_ concentration, and X_5_ is soil water content (Table 5).

**Table 5:** Multiple regression analysis of environmental factors and photosynthetic rates of soybean in different treatments in 2012.

Treatments	Multivariate stepwise regression equation	Multiple correlation coefficient (R^2^)
Control	Y = 1.7044+0.1289X_1_-0.1806X_2_	0.2856**
Poplar	Y = 8.2534+0.3873X_1_+0.2109X_2_-0.1487X_3_	0.5116*
Maple	Y = 10.0463+0.5556X_1_+0.3382X_2_+0.1996X_4_+0.0795X_5_	0.3377*
Walnut	Y = 11.6122+0.3417X_1_+0.1194X_4_	0.6889*

*, ** indicate significant at 5% and 1% levels, respectively.

Note: Y is the predicted value of NA, X_1_ is PAR, X_2_ is air temperature, X_3_ is RH, X_4_ is CO_2_ concentration, and X_5_ is soil water content.

PAR (X_1_) is highly correlated (p<0.01) with soybean NA in all intercrop treatments and less so in the control treatment (p<0.05). Atmospheric temperature (X_2_) is another relatively frequent impact factor in addition to photosynthetic active radiation (X_1_) in the given equations (Table 5). However, based on the F test (not shown), PAR had greater positive effect on NA rate than the atmospheric temperature. Atmospheric CO_2_ concentration (X_4_) had a positive effect on the NA of soybeans only in the maple and walnut treatments. On the other hand, relative humidity (X_3_) had a negative correlation with the NA rate of soybeans in the poplar treatment and the soil water content (X_5_) had a positive correlation on net assimilation rate only in the maple treatment.

## Discussion

Plant photosynthesis is a complicated physiological and biochemical process, and it is restricted by many environmental factors such as PAR, atmospheric temperature, atmospheric humidity and atmospheric CO_2_ concentration. In our study, intercropping systems were shown to improve field microclimatic conditions (e.g. reduced atmospheric temperature, increased RH). Given climate change conditions and associated climate extremes, in a dry year, enhanced RH above the understory crop canopy and lower atmospheric temperature may contribute to less evapotranspirative loss from the understory crop. However, PAR and CO_2_ concentrations measured in the intercropping systems were significantly lower than the same parameters measured in the monocropping system (control). As these two parameters had a positive correlation with the net assimilation (NA) of soybeans, this negatively influenced the final soybean above ground biomass and crop yield (Table 2). Similar results and observations have also been reported in other tree-based intercropping experiments [11–15].

The diurnal variation of NA of soybean in the monoculture system was a bimodal curve in our study and an obvious “lunch break” phenomenon was observed; however, in most intercropping treatments this phenomenon was not observed (Fig 3). In the control treatment, the NA was found to be lower during the peak PAR readings of the day (13:00) confirming that the “lunch break” phenomenon has a direct relationship with PAR [16]. However, Raschke and Resemann [17] studied the leaves of *Arbutus undeo* L. and showed that the photosynthetic “lunch break” phenomenon can occur at low levels of photosynthetically active radiation (about 500μmol m^-2^ s^-1^), and that it has a certain relationship with plant growth rhythm and genetic characteristics. Nevertheless, strong PAR is the basic driving force that causes a variety of changes in environmental factors such as reduced atmospheric humidity and increased atmospheric temperature, indirectly leading to the photosynthetic “lunch break” phenomenon [16]. In our study, the temperature in the monoculture soybean experimental plots was as high as 36.57°C (Table 4); this temperature could have caused adverse effects on the photosynthetic ability of soybean causing enhanced transpiration, leading to the photosynthetic “lunch break” phenomenon. Interestingly, the significantly lower ambient temperatures measured in all three intercropping treatments (Table 4) did not likely affect the photosynthesis process of the associated understory crop. The moderation of extreme temperatures often seen in tree-based intercropping systems is an important factor. Given the climate extremes that is being experienced and associated with crop losses, integration of trees into agricultural systems may enhance adaptation to climate changes [9] and reduce crop losses. While proper management strategies, such as branch pruning, is adopted to allow more PAR.

Soil water content (0–20 cm) of the intercropping system was significantly lower than that of the monoculture system. The shorter the distance from the trees, the stronger the water competition was between overstory trees and understory crops (Table 3). Reynolds et al. [10] found average soil moisture content to be 6.8% in the intercropping treatments on the same site when trees were only 12-year-old. The current study indicated the average soil moisture under intercropping treatments to be 5.6%. Therefore, it appears that competition for soil water under matured tree-based intercropping systems is unavoidable.

In our study, compared with the control treatment, the PAR under poplar and maple intercropping systems was reduced by 53.6% and 57.9%, respectively. In the 1997 study, Reynolds et al. [10] reported that PAR on soybeans under the poplar and maple intercropping systems was only reduced by 17.9% and 14.6%. In the same study, soybean seed yield was also reduced by 42.1% and 35.5%, respectively (Table 2), but in our study, seed yields were reduced by 71.8% and 64.4%, respectively. It is obvious that the growth of trees since 1997 (Table 1) has resulted in increased competition for light and soil water. However, the competition for light and soil water can be reduced with proper design and management such as adopting branch and lateral tree root pruning [9]in tree-based intercropping systems. Working with temperate tree-based intercropping systems in China, Wu and Zhu [18] have recommended that spacing be dependent upon the relative value of the crop and the tree. They recommended 5–10 m spacing where wood from the tree is the most valued, 15–20 m where both crops and trees are equally valuable (similar to the tree spacing in this study), and 30–50 m where the understory agricultural crop is the most valuable component. Based on their research results, the distance between trees (15 m), as in our study, was the correct distance between the tree rows. However, our research results are indicating that when trees get mature (see Table 1, from 1997 to 2012), a 15 m tree-row spacing may not be the right distance for understory agricultural crops (soybean). Therefore, the tree-row distance or spacing should be widened to reduce light and soil water competition and thereby maintain economic crop yields. Changing the design configuration by removing alternate trees within rows, or even removing alternate tree rows, should help to reduce competition for light and soil moisture. Another important factor to consider is the choice of the agricultural crop (C_3_ versus C_4_) to be grown in tree-based intercropping systems. As plants with a C_4_ photosynthetic pathway are less adapted to shade than those with a C_3_ pathway, C_4_ understory crops should be avoided under mature tree-based intercropping systems [19]. Newman et al. [13] reported that maize yields were reduced by 32% relative to monoculture crops when intercropped with *Paulownia fortunei* during the summer in China, whereas the yield of ginger was 34% greater than in monoculture crops. Similar results have also been reported for ginger by many other researchers [20, 21]. These observations suggest that reasonable crop yields in tree-based agroforestry systems might be maintained if correct shade-tolerant C_3_ species are utilized.

## Conclusions

It appears that when trees mature competition for moisture and light may become as limiting factors for crop growth. In 1997, when the trees were only 12-year-old, the average soil moisture measurement was 6.8% in the intercropping treatments [10]. The current study was conducted in 2012 and trees are now 26-year-old and the average soil moisture under intercropping treatments was 5.6%. When compared with the control treatment (no trees), the PAR under poplar and maple intercropping systems was reduced by 53.6% and 57.9%, respectively. In the 1997 study, at the same experimental site, the PAR on soybeans under poplar and maple intercropping systems was only reduced by 17.9% and 14.6%, respectively [10]. In the same study, soybeans seed yield was also reduced by 42.1% and 35.5%, respectively, but in our current study the yield was reduced by 71.8% and 64.4%, respectively. It is therefore suggested that for long-term benefits, thinning of trees and/or removing alternate trees rows and cultivation of shade tolerant crops may help to reduce light competition and possibly the competition for soil moisture. These results could be used as guidelines for improving and optimizing productivity in maturing agroforestry systems.
